# The intelligent engine start-stop trigger system based on the actual road running status

**DOI:** 10.1371/journal.pone.0253201

**Published:** 2021-06-25

**Authors:** Xinhuan Zhang, Hongjie Liu, Chengyuan Mao, Junqing Shi, Guolian Meng, Jinhong Wu, Yuran Pan

**Affiliations:** 1 The Institute of Road and Traffic Engineering, Zhejiang Normal University, Jinhua, Zhejiang Province, China; 2 School of Electronic and Information Engineering, Xi’an Jiao Tong University, Xi’an, Shanxi Province, China; Vellore Institute of Technology: VIT University, INDIA

## Abstract

With the rapid development of urbanization and the popularization of the vehicle, the frequent occurrence of traffic jams results in idling fuel waste, environmental pollution, and other issues. In order to alleviate these problems, engine start-stop technology has been widely used in different types of vehicles in recent years. However, current start-stop trigger technology has many deficiencies, such as mistaken triggering and frequent engine start-stop, which greatly reduces user driving experience, causing most of them to deactivate this system. The intelligent engine start-stop trigger (IEST) system based on the actual road running status was established by building the image recognition model and the digital traffic analysis model in order to solve this problem. A system test shows that IEST can avoid frequently engine starting and stopping. The results show that IEST could effectively improve the driving experience and reduce engine fuel consumption, and it promotes conventional engine start-stop technology.

## I. Introduction

With the rapid growth of automobile ownership, traffic congestion occurs frequently, which leads to the decrease of the capacity of road and intersections, and also increases the idle working time of automobiles, which not only leads to the increase of fuel consumption, but also aggravates the degree of environmental pollution. Therefore, the technical methods to make the cars pass the road and intersections efficiently and safely have always been a concern of people.

The working principle of an engine’s start-stop function of is: when the preset condition of stop is met, the engine automatically stops while the engine is idling, when intent to start driving or other engine requirements are detected, the engine will quickly start so as to reach normal working status [1, 2]. The engine start-stop function can effectively reduce the fuel consumption of the vehicle in idle status and reduce harmful gas emissions [3–5]. Therefore, research on engine start-stop control systems is of great significance.

Traditional engine start-stop technology’s working principle is that the engine stops once the brake pedal has been depressed for 2 seconds, and runs again when the brake pedal is depressed again, which helps save energy. However, this trigger technology has two important disadvantages:

1. When a vehicle stops for red light for less than 5 seconds, the fuel consumed by activating the engine start-stop technology is more than when the engine idles for a time for the red light.
2. It only considers the vehicle status, stopping or running, but neglects the road status, especially road congestion, which leads to frequent start-stop activation, further affecting both vehicle stability driving comfort.

The main reason for the above disadvantages is the unintelligent engine start-stop system trigger. To solve this problem, this paper combines the traditional engine start-stop system with intelligent identification of the road’s actual state, constructing image recognition module and a digital traffic analysis module. This paper proposes a new trigger mode for engine start-stop systems by judging the situation of the vehicle in congested traffic or stopping at a red light. These two modules could control an engine’s stopping time, thereby avoiding unnecessary engine start-stop. System testing proved that IEST can effectively improve the driving experience, reduce engine fuel consumption, and help promote traditional engine start-stop technology.

## II. Literature review

The general idea for an engine start-stop system is to save energy and support the driver in concentrating his or her attention on driving. In recent years, engine start-stop technology has been widely used in different types of vehicles [6–8].

In recent years, researchers taking advantage of one of the Artificial Intelligence (AI) fields [9] have proposed a lightweight and real-time traffic light detector for the autonomous vehicle platform. The model consists of a heuristic candidate region selection module to identify all possible traffic lights, and a lightweight Convolution Neural Network (CNN) classifier to classify the results obtained. With the number of cars increasing, how to coordinate the traffic light controllers of multiple intersections becomes a key challenge for multi-agent reinforcement learning (MARL). Most existing MARL studies are based on traditional Q-learning, but its unstable environment leads to poor learning in the complicated and dynamic traffic scenarios. Wu, Tong, et al. propose a novel multi-agent recurrent deep deterministic policy gradient (MARDDPG) algorithm based on the Deterministic Policy Gradient algorithm for traffic light control (TLC) in the vehicle networks [10]. Liang, Xiaoyuan, et al propose a deep reinforcement learning model to control the traffic light cycle [11]. The model quantifies the complex traffic scenario as states by collecting traffic data and dividing the whole intersection into small grids. The duration changes of a traffic light are the actions, which are modeled as a high-dimension Markov decision process. The result is the cumulative waiting time difference between the two cycles.

Many researchers have already investigated engine start-stop systems and the identification of traffic signals. For example, David Ibarra researched noise emissions of this system [12], and A. de la Escalera researched traffic sign recognition and analysis for vehicles [13]. Hirabayash proposed a method to recognize the state of traffic lights in images [14]. Lucas C. proposed to integrate the power of deep learning-based detection with maps previously used to recognize relevant traffic lights of predefined routes [15]. Jiankang Deng proposed a deep learning method to recognize faces [16]. HaoYang proposed efficient asymmetric one-directional 3D convolutions to approximate the traditional 3D convolution and achieve high performance in action recognition methods [17]. Cheng proposed a two-layer Convolutional Neural Network (CNN) to learn the high-level features which can effectively recognize the faces [18]. Haike developed an algorithm that can recognize traffic lights and dangerous driving events [19].

Due to digitization, a huge volume of data is being generated across several sectors such as Intelligent roadside equipment, IoT devices. Machine learning algorithms are used to uncover patterns among the attributes of this data. Not all the attributes in the datasets generated are important for training the machine learning algorithms. Some attributes might be irrelevant and some might not affect the outcome of the prediction. Thippa Reddy Gadekallu et al propose two of the prominent dimensionality reduction techniques, Linear Discriminant Analysis (LDA) and Principal Component Analysis (PCA) which can help ignoring or removing these irrelevant or less important attributes reduces the burden on machine learning algorithms [20]. Praveen Kumar Reddy Maddikunta et al propose that a machine learning based model implementing a random forest regression algorithm is used to predict the battery life of IoT devices. Several pre-processing techniques like transformation and dimensionality reduction are used in this model [21].

Because the starting and stopping of the brake involves the safety of the vehicle, Connected and Automated Vehicles (CAVs), the sensor-generated data are, however, vulnerable to anomalies caused due to faults, errors, and/or cyberattacks, which may cause accidents resulting in fatal casualties. To help in avoiding such situations by timely detecting anomalies, Abdul Rehman Javed et al propose an anomaly detection method that incorporates a combination of a multi-stage attention mechanism with a Long Short-Term Memory (LSTM)-based Convolutional Neural Network (CNN), The MSALSTM-CNN method effectively enhances the anomaly detection rate in both low and high magnitude cases of anomalous instances in the dataset [22]. Abdul Rehman Javed et al propose a novel approach named CANintelliIDS, for vehicle intrusion attack detection on the CAN bus. CANintelliIDS is based on a combination of CNN and attention-based GRU model to detect single intrusion attacks as well as mixed intrusion attacks on a CAN bus [23].

At present, the engine start-stop control system is too simple to judge the stop conditions. Generally, it controls the stop when the engine stops idling, and lacks consideration of road conditions and environment. It is easy to cause frequent start-stop when there is no need to start-stop, and there is no start-stop function when there is need to start-stop, thus causing the problem of increased fuel consumption. For example, when the signal light is about to change from red to green (test data shows that the fuel consumption in one start is equivalent to 5 seconds of idling caused by the gasoline engine and 20 seconds of idling caused by the diesel engine [24]), during the waiting at the red light for less than a certain time, it is better to save fuel and reduce pollutant emissions without using the start-stop function. The frequent start-up and stop of the engine in the lightly congested road section and the failure of the air conditioning after the engine shutdown in high temperature weather cause bad driving experience for the driver and user. Some users even turn off the engine start-stop function for a long time. And because the starting and stopping of the brake involves the safety of the vehicle, it needs to set up high-precision special sensors for monitoring and protection, and needs to use a variety of strengthening components, so the development cycle is long, the production cost is high and the usability is poor, not suitable for general use. Therefore, it is very necessary to design and develop the control system and control method suitable for universal application based on the mature starter, battery and other components of the existing engine start-stop system.

In view of the fact that the current hardware development level of engine start-stop system has reached a certain level, but there are still some defects such as start delay, jitter, inaccurate judgment of shutdown and so on, it is possible to get twice the result with half effort to focus on the development of control system and control method suitable for popular application. At the same time, for the problem that the current engine start-stop control system cannot sense the actual running environment information of the road, based on the existing logic of engine start-stop control, an intelligent engine start-stop trigger system based on the actual road running state is proposed to make the engine start-stop control more accurate, so as to improve the fuel saving effect of the engine automatic start-stop function and the user driving experience. It can be predicted that the engine start-stop control system and control logic with remarkable energy saving and emission reduction effect and excellent user experience will become the current and future research and development hotspot.

## III. Methodology

**Fig 1:**
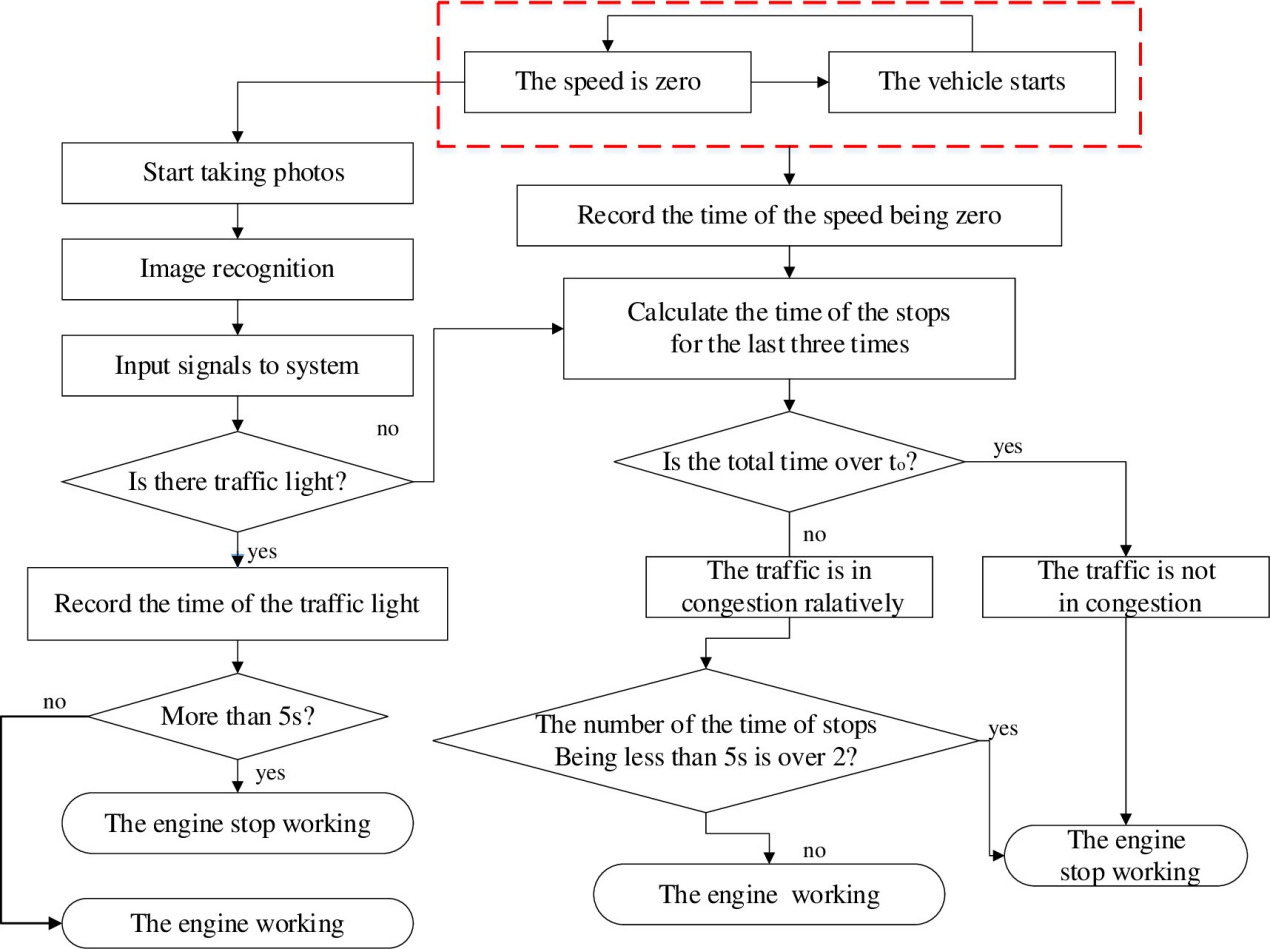
The flow chart of IEST working principle.

Fig 1 represents the IEST(The intelligent engine start-stop trigger system) working principle as a flow chart. When encountering a traffic light or traffic jam, an IEST-equipped vehicle would first check the state of all sensors and then determine whether the vehicle engine meets the traditional shutdown conditions. The traditional shutdown conditions are 1) The wheel speed sensors of the antilock system display zero. 2) The transmission is not in reverse gear. 3) Electronic battery sensors detect that the battery has enough energy for the next ignition [25]. With these conditions satisfied, IEST enables the image recognition module to work, and the camera starts to take pictures of the front road to obtain traffic light information. If the red light’s remaining time is more than 5 seconds, the engine will stop working. If the image recognition module does not detect a red light in the image, the digital traffic analysis module will start to judge whether the road is in congestion or not. By judging the road condition and the driver’s intention, IEST will give commands and instructions to control the engine.

After satisfying the traditional shutdown conditions, IEST will work the engine start-stop system with greater accuracy by identifying the road condition, which not only can maximize fuel economy and reduce CO_2_ emissions but also avoid the frequent vehicle start-stop common on congested roads and in turn improve driver willingness to use the engine start-stop system.

## IV. The recognition of traffic lights

A large amount of image-based data has recently become available in many disciplines, including medicine [10], agricultural production [26], geology [27], ecology [28], and also in the traffic domain. In this project, image processing technology was applied to identify the actual road status.

**Fig 2:**
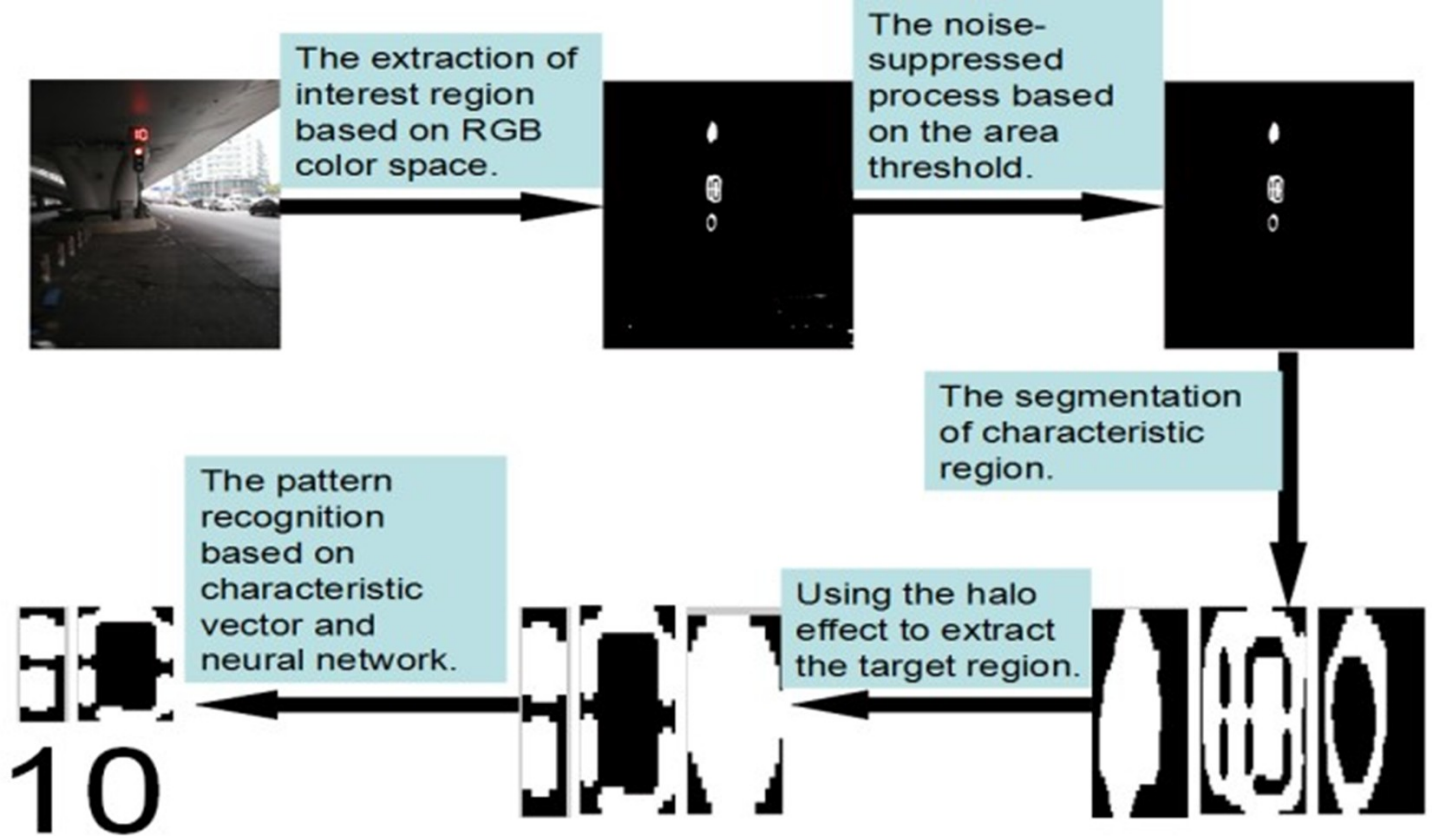
The flow chart of image recognition and image process.

The traffic signal identification process is shown in Fig 2. The details of how the system decodes and recognizes the image are presented in the following section.

### A. Extracting the characteristic region

#### 1) The extraction of the target region in the RGB space

Recently, various models of human upper-limb anatomy have been derived. The biomechanical models of the arm that represent precise anatomical models including muscles, tendons, and bones are too complex to be utilized in the mechanical design of an anthropomorphic robot arm. From the view of the mechanism, we should set up a more practical model for easy and effective realization. The RGB model has been widely used in the image recognition area [11, 29–31] but is rarely used for characteristic region division because RGB components are easily affected by light. However, the difference corresponded to three components from three colors remains at a certain range and the difference is hardly affected by the light, so the difference could be applied to make the characteristic region get better segmentation in RGB space without space conversion in real-time.

**Fig 3:**
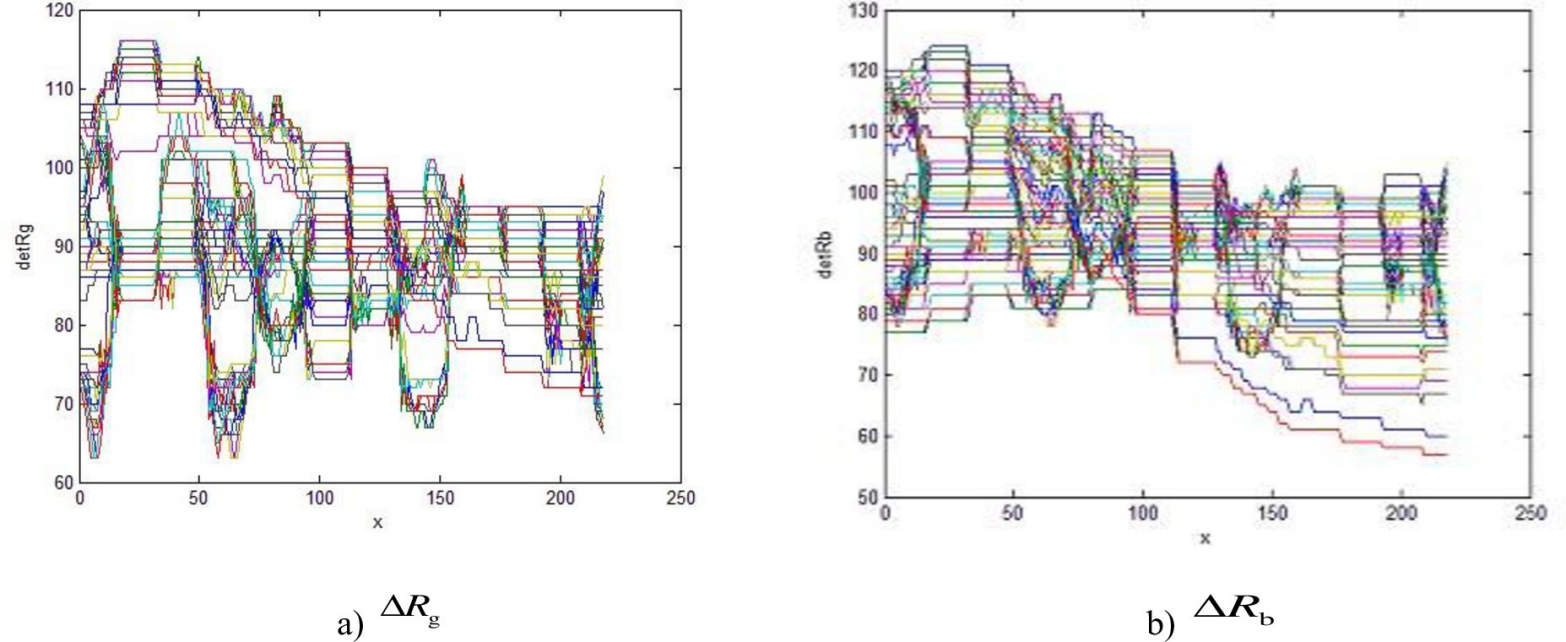
The red area distribution.

In this module, the key part of the image which needs to be identified is the red area, and the R, G difference marked as ΔR_g_, ΔR_b_, could be used as the judgment basis to extract the characteristic region. The distribution of ΔR_g_, ΔR_b_ between the red area and other color areas in the traffic light are shown in Fig 3.

Comparing Figs 3 with 4, the value ΔR_g_, ΔR_b_ of red areas is found to be larger than 60, while in other color areas the values are smaller than 20, so the difference ΔR_g_ and ΔR_b_, could be used to be the identification parameters to recognize the traffic lights. The equation can be expressed by Eq 1

$$\{\begin{array}{c} p_{ij}=1\;(R_{ij}-G_{ij})>60,(R_{ij}-B_{ij})>60\\ p_{ij}=0\;(R_{ij}-G_{ij})\leq 60,(R_{ij}-B_{ij})\leq 60 \end{array}$$ (1)

*R*_*ij*_ is the R value of the pixel in row *i* and column *j* in the original image. *G*_*ij*_ is the G value of the pixel in row *i* and column *j* in the original image. *B*_*ij*_ is the B value of the pixel in row *i* and column *j* in the original image.

**Fig 4:**
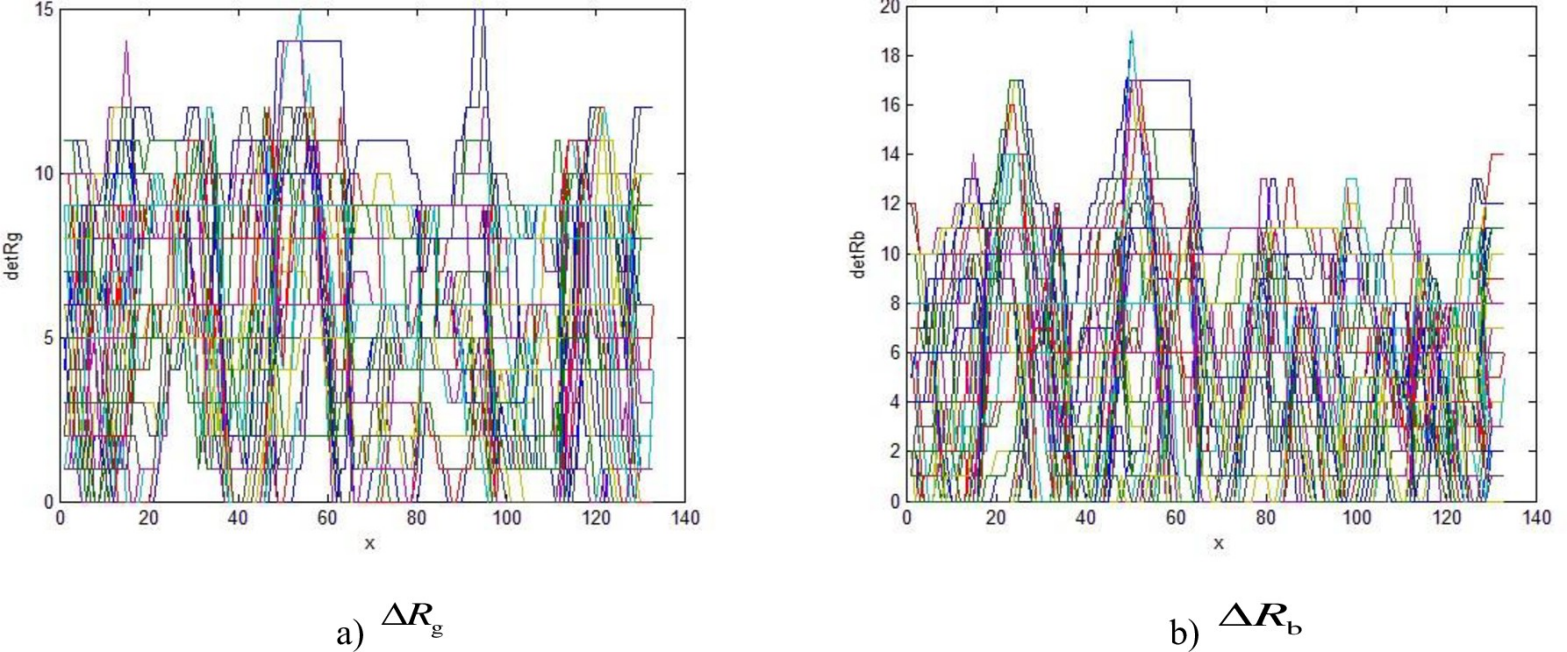
Other color area distribution.

*P* is the binary image. *P*_*ij*_ is the grey value of the binary image.

**Fig 5:**
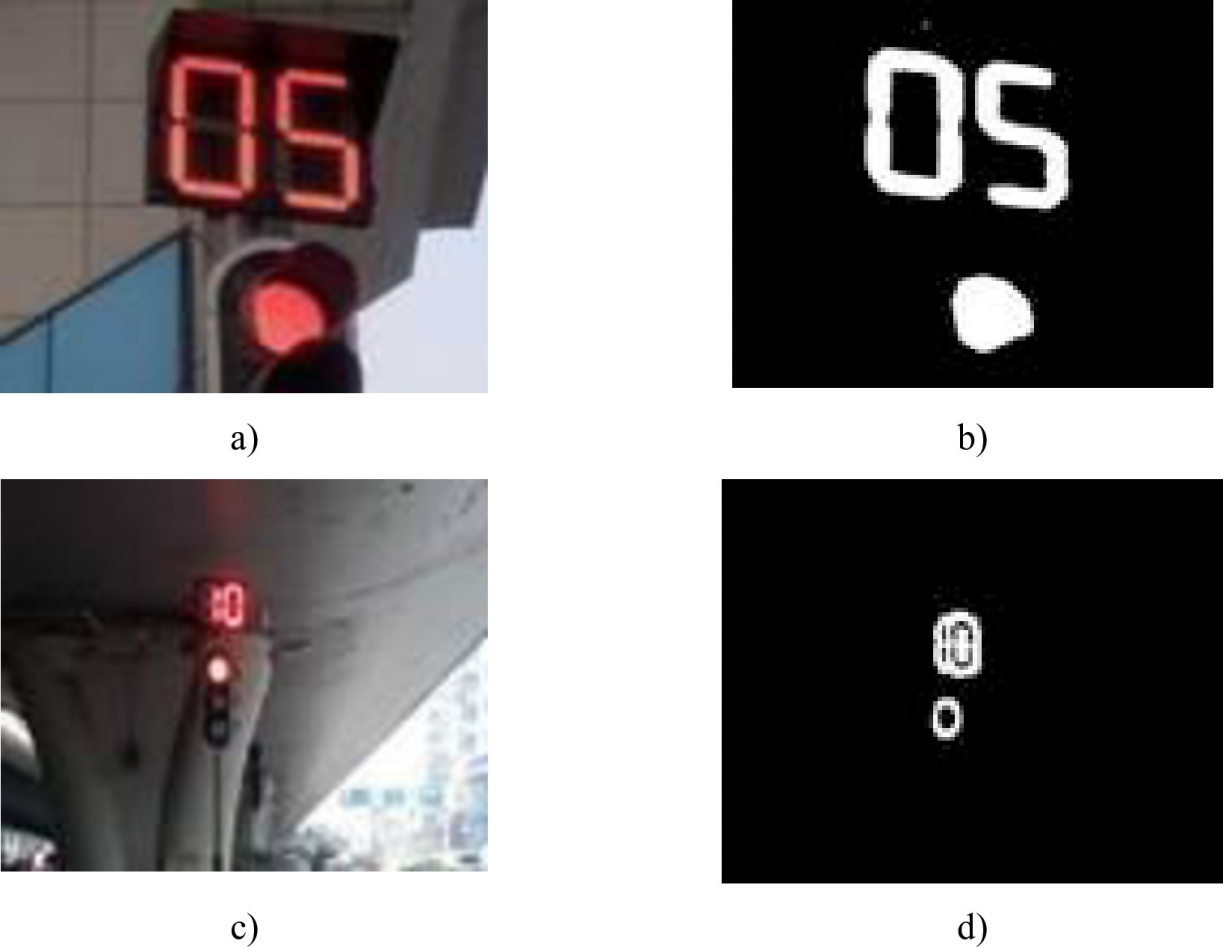
The comparison images before and after traffic lights color recognition.

The images after the color recognition process are shown in Fig 5.

The pictures in Fig 5 reveal that the RGB model could be applied to extract the common target region. However, some traffic lights with strong light intensity would cause a halo effect as shown in Fig 5(C). From this picture, the halo effect shows that the outline of the light is red while the lamp is white, which causes the characteristic region with the jagged white edge and the black inner part like Fig 5(D). The survey found that this phenomenon is very common. The halo effect increases the difficulty in extract the Target Region. Therefore, in order to solve this problem, a method for using the halo effect to extract the target region was studied in this paper.

#### 2) The extraction of the target region using the halo effect

The halo effect causes two different colors (the lamp color and the tube periphery color) to mix together and makes the target region fuzzy: the lamp is the inner layer of the target region with white color and the tube periphery is the outer layer with red color. In this research, the extraction of light color and the number is the most important part in this module. Therefore, the inner layer is the part required to be extracted. Based on the pixel difference experience threshold method, the outer layer can be successfully extracted in the first step. The next step is to extract the inner layer.

By analyzing the color recognition results, this module, through the character of the halo surrounding the inner layer, first extracts the outer layer edge and then negates the inner layer so as to extract the target region. The algorithm is expressed as follows:

**Step 1:** The whole image could be divided into *n* image matrices by the way of the traffic lights color recognition and the size of the *k-th* image matrix is *M*_*k*_ * *N*_*k*_, *k* = 1;
**Step 2:** Scan the column *j* in the *k-th* matrix named *p*, if *P*_*i*01,*j*_ < 0 and P_*ij*_ > 0, then *x*_1_ = *i*, *y*_1_ = *j* or else repeat step 2, when *i* = *M*_*k*_, then *j = j* + 1, if *j* = *N*_*k*_, go into step 5.
**Step 3:** Do *i* = *i* + 1, if *P*_*i(j−1)*_ > 0 and *P*_*ij*_ < 0 then *x*_2_ = *i*, *y*_2_ = *j* or else repeat step 3 until *i* = *M*_*k*_, do *x*_2_ = *M*_*k*_, *y*_2_ = *j*;
**Step 4:** Do *P*_*ij*_ = − (*P*_*ij*_ − 1), *i*∈(*x*_1_, *x*_2_), *j*∈(*y*_1_, *y*_2_), *j = j* + 1 and *i*, *j* are integer and next return to step 2;
**Step 5:** The matrix *p* should be divided as step 1, meanwhile do *k = k* + 1 and return to step 2, if *k = n*, the algorithm ends.

**Fig 6:**
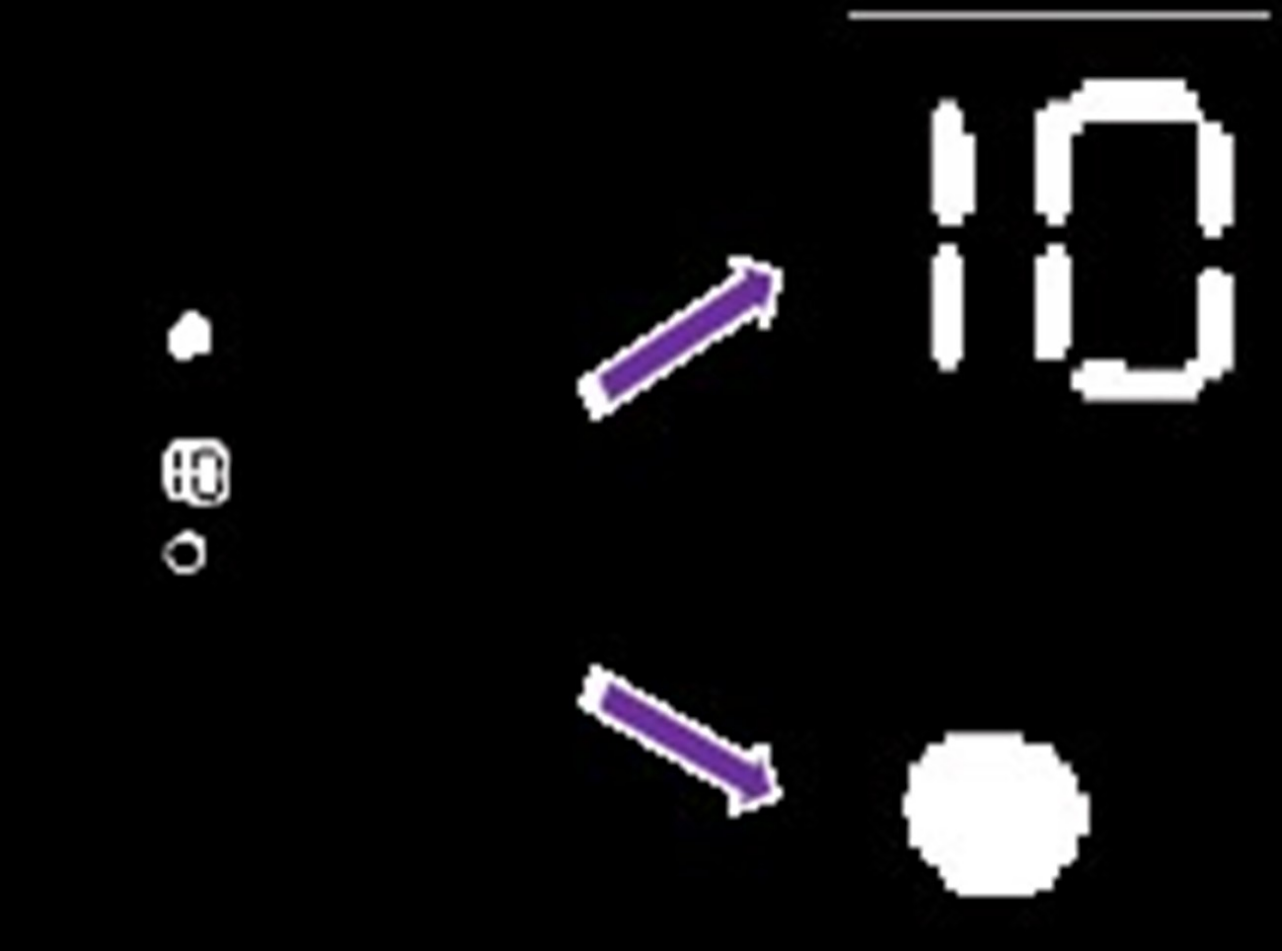
The extraction of the target region with the use of the halo effect.

Fig 6 shows the image when the halo effect (Fig 3(D)) has been processed by the above algorithm. The result shows that the image with the halo effect has been successfully transferred into the normal image (as shown in Fig 5(B).

### B. The noise-suppressed processing based on the area threshold

**Fig 7:**
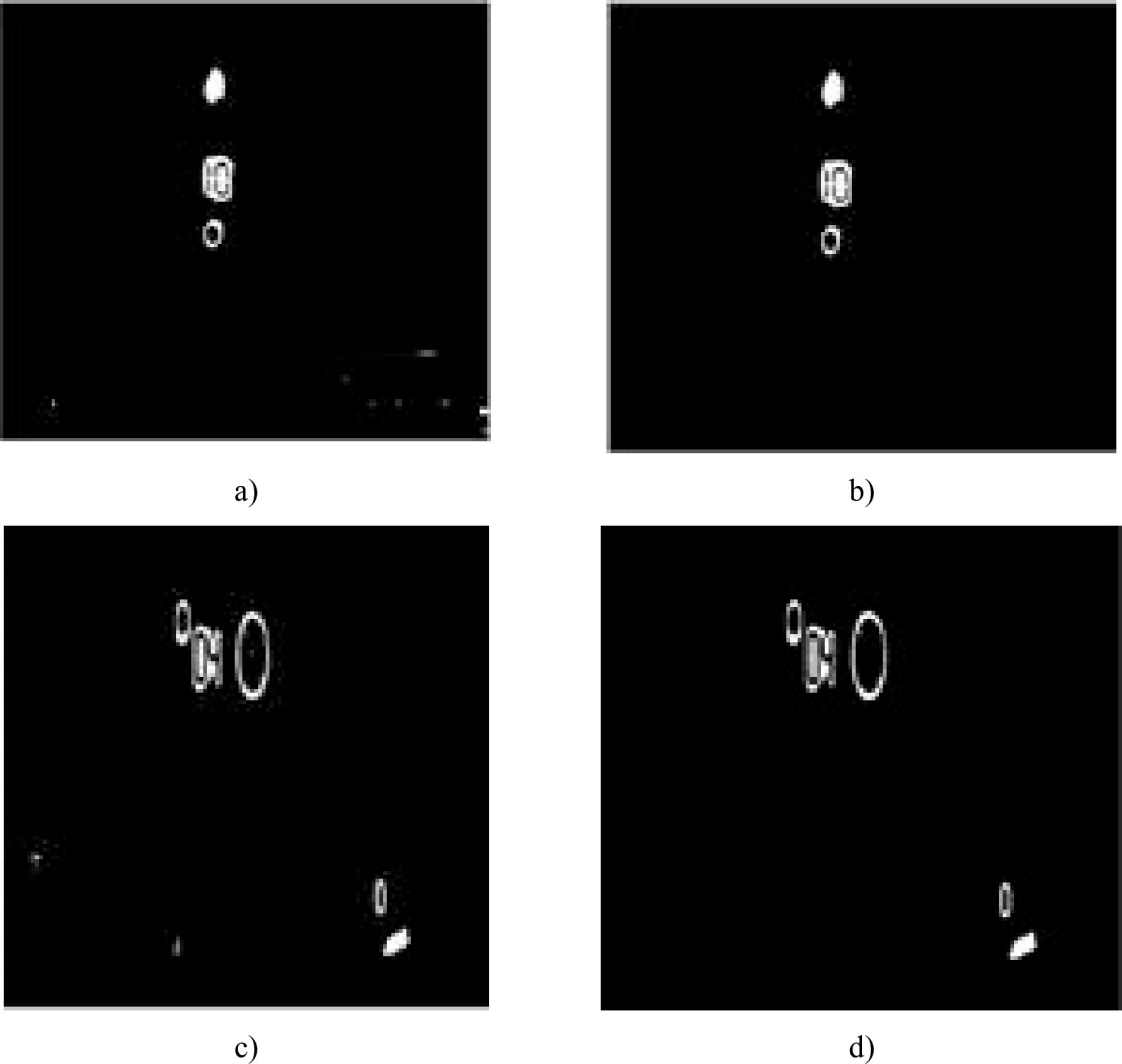
Comparison images before and after denoising.

Because there are some noise points which are similar to the target region, the noise points should be eliminated from the target region to make pattern matching easier. The noise points are easily removed because the noise-connected area is generally smaller than the target region [32]. As shown in Fig 7, most of the noise points have been removed and the large noise area (shown in the lower left area in Fig 7(C)) can also be removed during pattern recognition.

### C. The characteristic region segmentation

Before conducting the pattern recognition, the target region must be divided with the projection method to improve the matching speed. The projection segmentation process consists of the following steps:

**Step 1:** Complete the vertical projection of the image.
**Step 2:** Record the number of white points in each column.
**Step 3:** Retain the part that the number of white points of each column is over 3 and remove the part that the number of white points of each column is less than 3.

Given that the size of the picture is *M* * *N*, the projection method is shown as the following formulas:

$$\left\{ \begin{array}{l} S(j)=\sum_{j=1}^{M}p_{ij}\\ j=2,3,4∼N,\mathrm{if}S(j-1)>3,\;thenS(j)>3,j=j^{'} \end{array}\right.$$ (2)

The value *J* could be divided into *k* sets and the length of *k-th* set indicated as *C* is *n*_*k*_. The element *J*_*kj’*_ in *J*_*k*_ should obey the following formulas:

$$\left\{ \begin{array}{l} J_{kj^{'}}=J_{k(j'-1)}+1\;\mathrm{where}:j^{'}=1,2,3∼n_{k}\\ S(J_{kj^{'}})>3 \end{array}\right.$$ (3)

**Fig 8:**
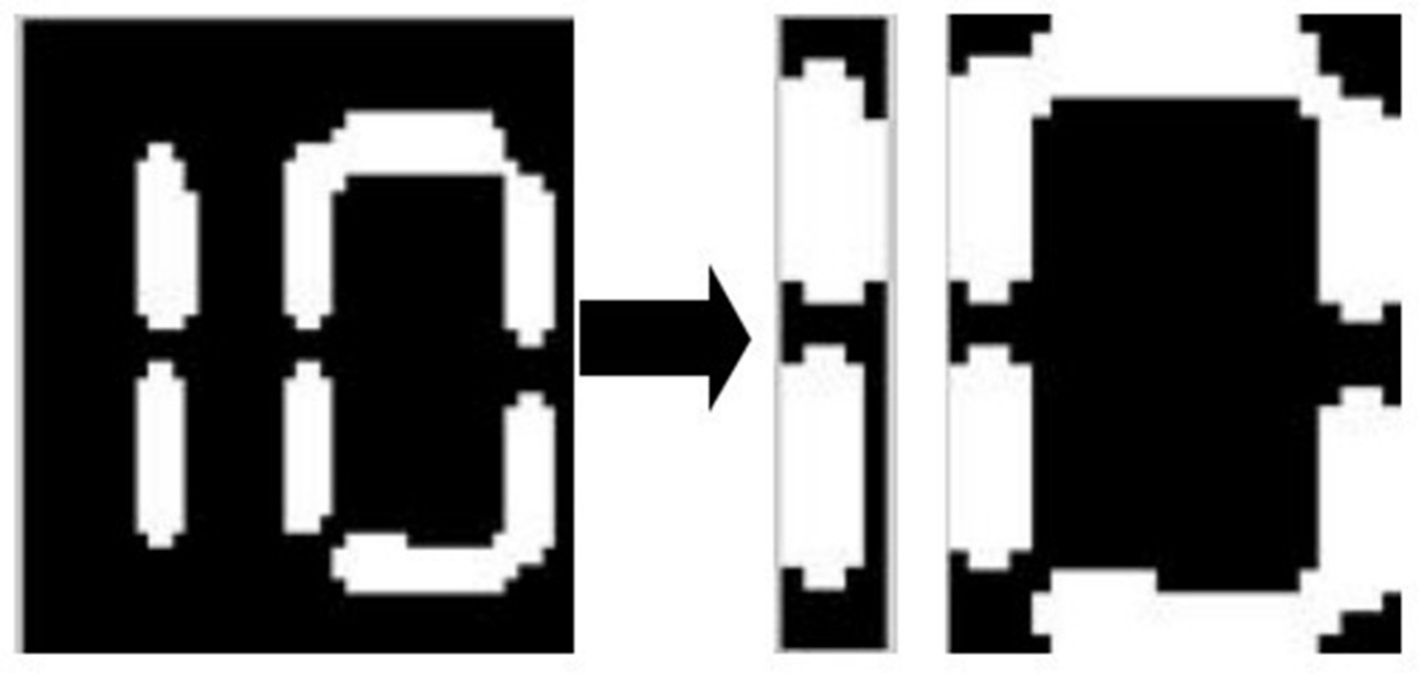
The image segmentation based on the projection method.

Fig 8 shows the image divided by the projection method, the result is shown in Fig 8.

### D. The pattern recognition based on characteristic vector and neural network

#### 1) The extraction and selection of characteristic

Different objects appear on images and these objects should be classified, recognized, or identified. Object classification can be effectively accomplished through the extraction and selection of characteristic quantities [27]. The characteristic quantity should not be affected by object movement that includes image translation, rotation, and scaling [33].

Hu, under the rule of definition, describes the characteristics of scale invariance, translation invariance, and rotation invariance [27, 34, 35]. The binarized image could be expressed with the two-dimensional density function, therefore the invariant moment could be applied to analyze the image characteristic. For the two-dimensional image, seven invariance moments, φ1, φ2…φ7 are the characteristic values.

To recognize traffic light time, the characteristic value of the templates from the numbers 0 to 9 plays an important role in the matching pattern process. Due to the influences of light and shooting angle, seven characteristic values of the binary image of the number a (a = 0,1,2… 9) are affected. In order to improve the identification accuracy, in this research, thirty characteristic values (*η*_*i*,1_ ∼ *η*_*i*,30_, *i* = 0…7) of each number a (a = 0,1,2… 9) have been obtained and first the false value should be eliminated from these values according to the error processing criterion. Then the average value of the remaining *η*_1,*j*_ must be calculated as the template characteristic value *η*_1_. Similarly, *η*_2_, *η*_3_, *η*_4_, *η*_5_, *η*_6_, *η*_7_ could be also regarded as template characteristic values.

#### 2) Pattern matching based on neural networks

Neural Networks (NNs) have been widely used in pattern recognition, function approximation, and many other application areas in recent years and have shown their strength in solving hard problems.

This module applied neural network technology in order to improve the accuracy of the pattern matching process. Formally, a neural network consists of Q layers, where the first layer denotes the input, the last one, Q is the output, and the intermediate layers are hidden layers. Since the seven invariant moments were used as the image characteristic values, the input node *m* is 7 and the total number of categories *a* is 11 and the output layer nodes *n* is 11. The number of nodes in the hidden layer could be determined by Eq 4:

$$n_{1}=\sqrt{m+n}+a$$ (4)

**Fig 9:**
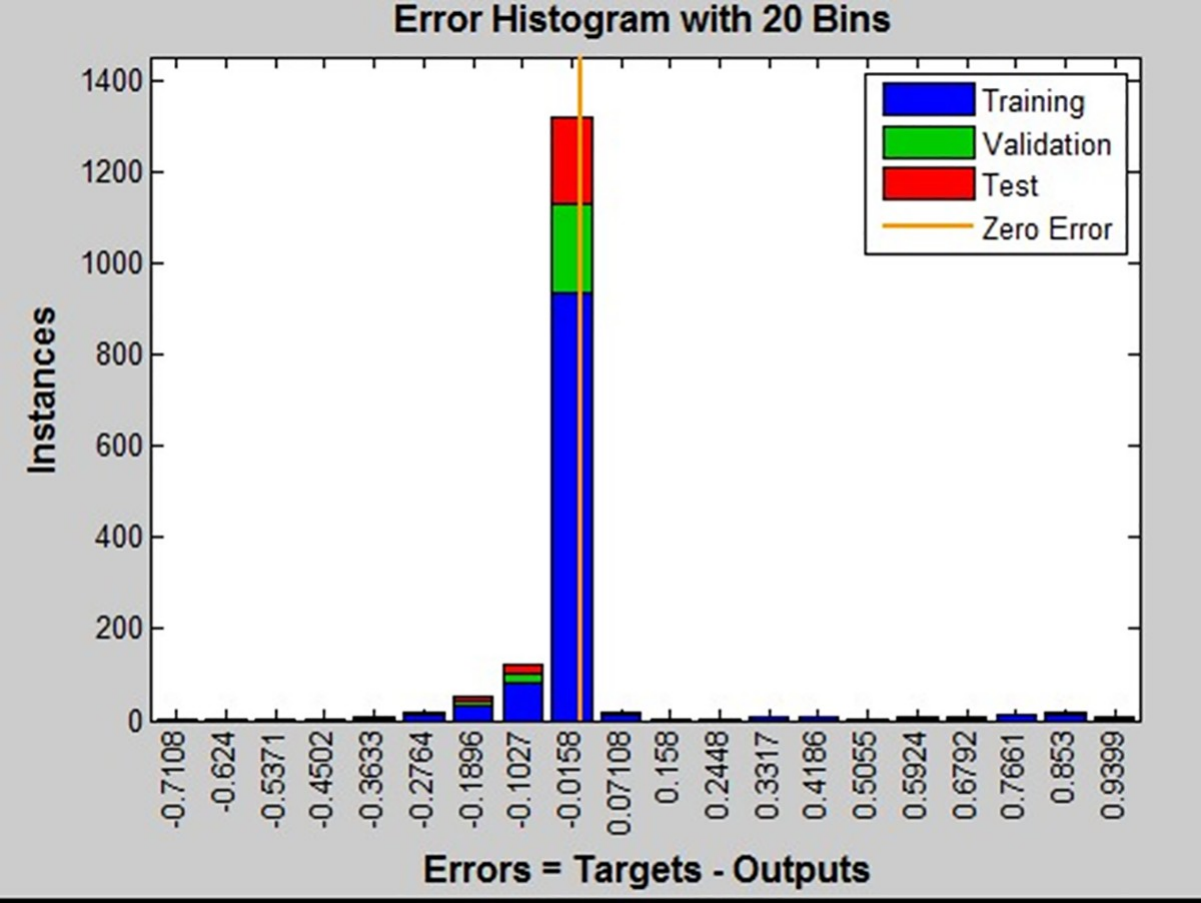
The gradient normalization errors of these samples.

*n*_1_ is the number of nodes in hidden layer, *n*_1_ = 15 in this paper. This module takes the TRAINSCG conjugate gradient method as the new weight learning algorithm, and 147 samples were tested for neural network training. The gradient normalization errors of these samples after 49 iterations and gradient normalizations. are shown in Fig 9.

From Fig 9, the neural network test errors are concentrated at -0.0158, which indicates that the neural network could be used in traffic lights template matching. Using this method, the rate of recognition accuracy among 90 traffic- intersection-pictures taken in this project is more than 90%.

## V. The analysis of traffic flow

### A. The theoretical analysis of road congestion judgment

This module applied statistical principles to analyze and judge the road conditions in order to alleviate the frequent rate of engine start and stop during the rush hour.

According to queuing theory in operational research, given a single random arrival of vehicles, the average arrival rate is *λ* and the average arrival interval between two cars is 1/*λ* at a certain distance. The departure rate is *μ* in one single lane, so the average service time is 1/*μ*. The ratio *ρ* = *λ/μ* is called the traffic intensity factor used to determine the road various status. When *ρ* < 1(*λ < μ*) and time is enough, each status (the jammed status, the semi-jammed status, the smooth flow status) of this road would be recurring. When *ρ* > 1, each status is unstable and the length of the line becomes longer with no upper limit. Thus, the condition to maintain a stable state that could ensure a single-channel queuing evacuation is *ρ* < 1. The study shows that when traffic intensity *ρ* is over 1.1, queue length will be increase rapidly and the service level decline rapidly [36].

This study observed many city roads while considering the principle just described. One representative road with *A* and *B* intersections was chosen for presentation here. Table 1 shows statistics for the same period of 10 working days:

**Table 1:** The statistics of a road vehicle state.

Counting Times	Time	Number of large cars(A)	Number of small cars(A)	Average arrival rate(A)	Number of large cars(B)	Average arrival rate(B)	Diverging rate(*μ*) (B)	Number of stops	Number of traffic lights	*ρ*
1	17:00–17:30	143	1520	1663	144	1524	1668	0	0	1.001
2	17:30–18:00	82	846	928	70	736	806	73	0	1.151
…	…	…	…	…	…	…	…	…	…	…
20	17:30–18:00	68	778	846	58	712	760	84	0	1.116

**Fig 10:**
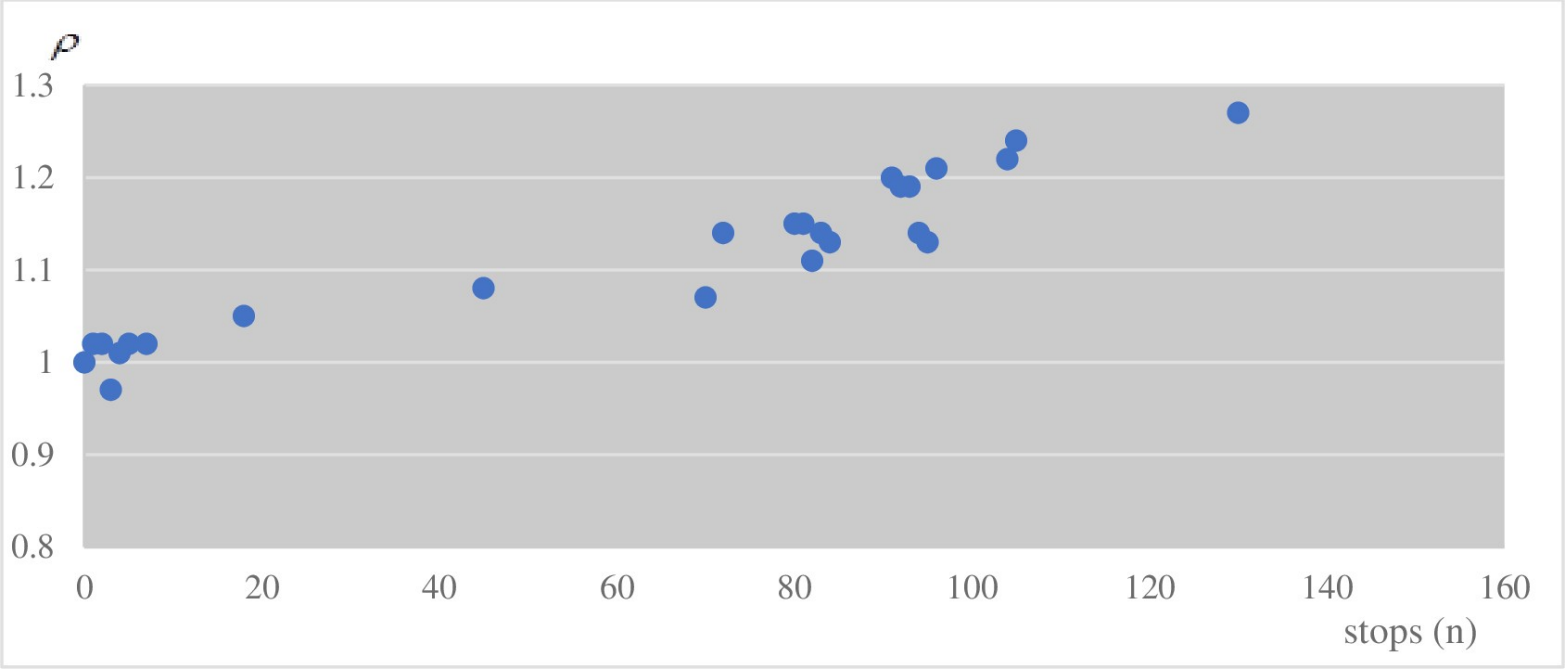
The relation between *ρ* and the number of the car stops.

Fig 10 shows a scatter diagram made from twenty groups of measured data (from Table 1) using the error processing criterion to rule out accidental values.

From Fig 10. *ρ* is directly interrelated with the number of cars stops, therefore *ρ* is regarded as the reference to judge road conditions. Moreover, the time of car stops per unit time is another measure of road congestion.

### B. The basic principles of traffic judgment

When *ρ* is over 1.1, this system considers that the road condition is in congestion. From Table 1, the stops frequency *f* is 3 during 58.536 seconds. Hence, if the number of stops is over 3 times during t_0_ seconds (t_0_ = 58.36s), the road is in congestion in this system.

When this module works, it records the time of the latest three stopping and starting of the vehicle, then calculates and analyzes the total time of these three stops. If the total time is less than t_0_ and two stopping duration is less than 5s, the engine would stop. If the total time is over t_0_, this system is turned off by default according to the basic principles of traffic judgment.

Since a car mainly stops in traffic jams or the traffic light in city roads, this system could save fuel and improve the driving experience.

## VI. System test

**Fig 11:**
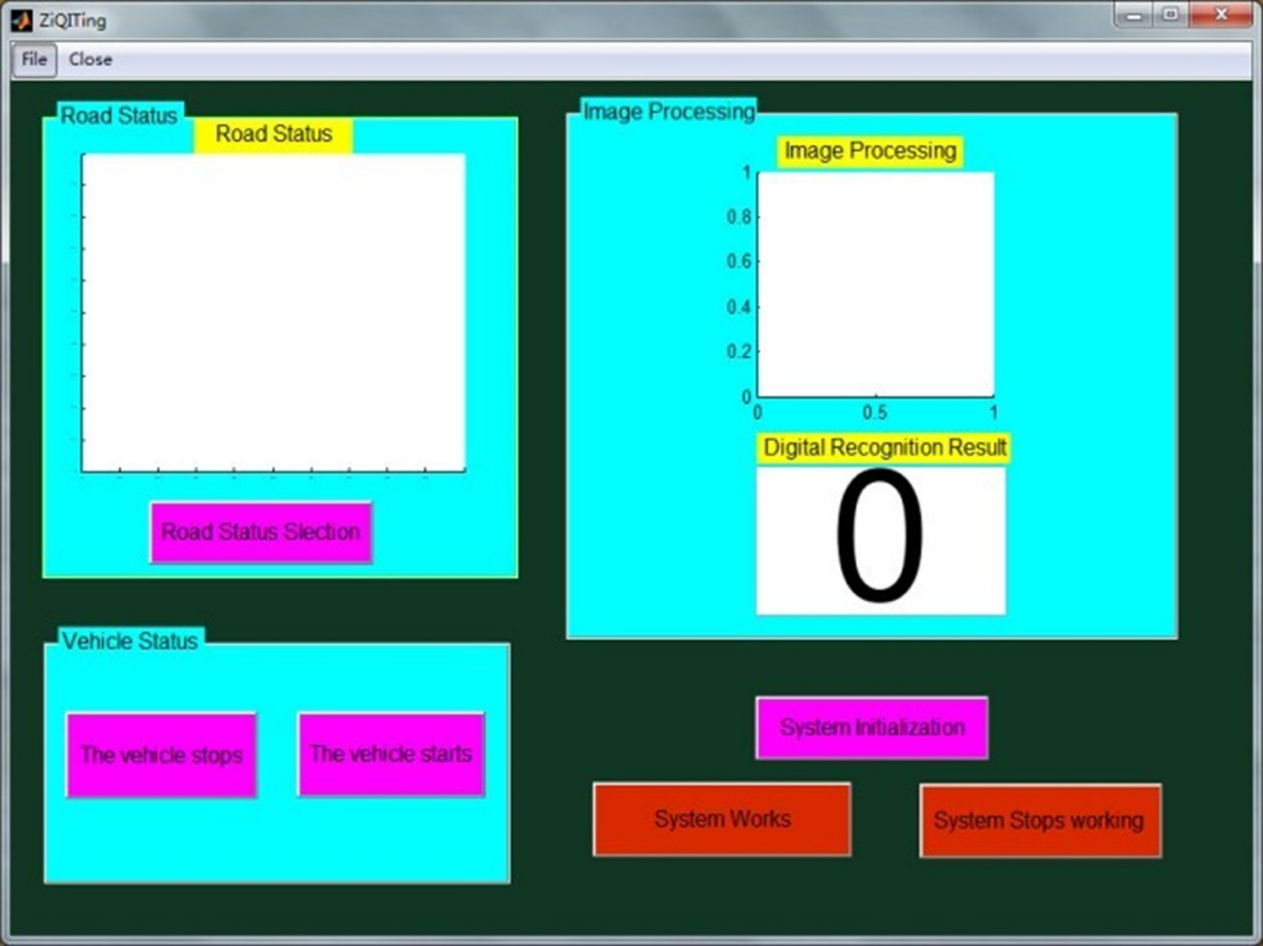
The software interface of IEST.

The intelligent engine start-stop trigger system software based on MATLAB was designed according to the image recognition module and the digital traffic analysis module this paper, describes. Fig 11 shows the software interface.

The software includes simulations of road status and vehicle status. When the trigger system is activated, each step of the image processing shows on the interface. The final result determining whether or not the engine works will appear on the interface. This study tested many kinds of road status. Fig 11 shows the test processing for typical road statuses.

✓ When red light time remaining is over 5s, the engine will stop, or else it will not stop.
✓ If a road status has no traffic lights but is congested, the digital traffic analysis module will activate to detect the road status and determine whether the engine must stop.

These results are consistent with the principle of the IEST system, and from system testing conducted in this study, the IEST with the described software platform has been determined to work correctly under these road statuses. It also shows that the theory underlying IEST is correct and feasible.

## VII. Conclusion

This paper proposed a novel IEST system to reduce unnecessary stops by adopting image recognition technology and numerical statistical techniques that can judge road condition. This paper also did study the system’s ability to judge road condition and establish a reference of congestion evaluation. Overall, this system is of great significance to further promote the traditional engine start-stop system so as to achieve the aims of energy savings and lower CO_2_ emissions.
